# Antifungal Action of Herbal Plants’ Glycolic Extracts against *Candida* Species

**DOI:** 10.3390/molecules28062857

**Published:** 2023-03-22

**Authors:** Vanessa Marques Meccatti, Lana Ferreira Santos, Lara Steffany de Carvalho, Clara Bulhões Souza, Cláudio Antonio Talge Carvalho, Maria Cristina Marcucci, Amjad Abu Hasna, Luciane Dias de Oliveira

**Affiliations:** 1 Department of Biosciences and Oral Diagnosis, Institute of Science and Technology, São Paulo State University (ICT-UNESP), São José dos Campos 12245-000, SP, Brazil; 2 Department of Restorative Dentistry, Endodontics Division, Institute of Science and Technology, São Paulo State University (ICT-UNESP), São José dos Campos 12245-000, SP, Brazil

**Keywords:** *Curcuma longa*, *Rosa centifolia*, Rosmarinus, *Punica granatum*, *Candida albicans*, *Candida dubliniensis*, *Candida tropicalis*, *Candida krusei*

## Abstract

*Candida* spp. cause fungal infection that affects patients’ oral health. This study aimed to evaluate the isolated and synergistic antifungal effect of *Rosa centifolia* L., *Curcuma longa* L., *Rosmarinus officinalis* L., and *Punica granatum* L. glycolic extracts against *Candida albicans*, *Candida dubliniensis*, *Candida tropicalis*, and *Candida krusei* planktonic and biofilm forms. The plant extracts were chemically characterized and the main compounds were quantified by high-performance liquid chromatography (HPLC-DAD) analysis. The minimum inhibitory and minimum fungicidal concentrations of the extracts were determined, and antibiofilm activity was evaluated by MTT assay. Data were analyzed by one-way ANOVA and Tukey’s tests, and by Kruskal–Wallis and Dunn’s tests, considering a significance level of 5%. The main compounds identified in each of the extracts were: p-coumaric acid (2153.22 μg/100 mL) in the rosemary extract, gallotannins (4318.31 μg/100 mL) in the pomegranate extract, quercetin derivatives (3316.50 μg/100 mL) in the extract of white roses, and curcumin (135.09 μg/100 mL) in the turmeric extract. The combination of *R. centifolia* and *C. longa* glycolic extracts was effective against *C. albicans, C. dubliniensis*, and *C. tropicalis* biofilms over different periods (*p* < 0.05). The combination of *R. officinalis* and *P. granatum* glycolic extracts was effective against *C. albicans* and *C. krusei* biofilms after 30 min, and against *C. tropicalis* after 24 h, with all combinations showing an average reduction of 50% in cell viability (*p* < 0.05). In conclusion, the combined plant extracts have antifungal and antibiofilm action against *Candida* spp. in different concentrations and times of action.

## 1. Introduction

Fungal infection (mycosis) ranges from superficial, or subcutaneous, to life-threatening systemic infections, it affects immunosuppressed patients, such as acquired immunodeficiency syndrome (AIDS) and cancer patients [1], it also effects patients hospitalized with COVID-19, that have an increased risk of co-infection with opportunistic pathogens, as the severe acute respiratory syndrome coronavirus 2 (SARS-CoV-2) is endowed with strategies to deregulate immune mechanisms [2]. Several oral conditions were reported in COVID-19 patients, including fungal infections like oral thrush (oral candidiasis), particularly among those who already had a predisposing condition, and were treated with antibiotics [3]. Furthermore, *Candida* spp., especially *Candida albicans*, have a role in primary and secondary endodontic infections, acting as co-pathogens with bacteria [4], and in periodontal infections [5], in which sodium hypochlorite and chlorhexidine solution have limited antifungal activity [6].

Besides, oral candidiasis is a fungal infection of the oral cavity, caused by *Candida albicans* and other *Candida* species [7]. *Candida albicans* is responsible for 95% of oral candidiasis cases, it has a remarkable repertoire of virulence factors that facilitate its transition from a commensal to a pathogenic state, it has a high capacity for adhesion, biofilm formation, and antibody degradation [8]. The dimorphism is the essence of the pathogenesis of this species [9]. *Candida dubliniensis* is the most common non-*albicans* species detected in isolates from oral cancer patients [10], and *Candida tropicalis* is a species that has a high capacity for adhesion and biofilm formation [11]. *Candida dubliniensis* and *C. tropicalis* are non-*albicans* species that display a considerable increase in the oral cavity of elderly patients [12], making the oral health maintenance a challenge especially in dentures-wearing elderly patients. *Candida krusei* is yet another emerging fungal nosocomial pathogen, primarily found in the immunocompromised and those with hematological malignancies [13].

The increased use of fluconazole and other antifungal agents, has resulted in the emergence of more resistant strains of *Candida* species [14], highlighting the need to investigate new alternative therapeutics with antifungal action [15,16,17,18]. *Rosa centifolia* L. (*R. centifolia*), or hundred-leaved rose or *Shatapatri* or *Taruni* (Rosaceae family), is a perennial plant available throughout India [19], it has antifungal effects against *C. albicans* and *C. tropicalis* [20]. *Curcuma longa* L. (*C. longa*), or turmeric (Zingiberaceae family), is a flowering plant [21] that has antifungal effects against *C. albicans*, *C. glabrata*, *C. krusei*, and *C. tropicalis* [15,22]. *Rosmarinus officinalis* L. (*R. officinalis*), or rosemary (Lamiaceae family), is an ancient shrub originating from the Mediterranean region [23], it has antifungal action against *C. albicans, C. dubliniensis, C. glabrata, C.krusei,* and *C. tropicalis* in vitro, and in *Galleria mellonella* model studies [17,18,24]. *Punica granatum* L. (*P. granatum*), or pomegranate (Lythraceae family, Punicoideae subfamily), is widely used as an alternative medicine [25], it is effective against *Candida* spp. [26].

The positive outcomes of the above-cited studies encouraged us to investigate the combined effect of these herbal extracts, as this may increase the treatment efficacy and reduce microbial resistance possibility [27]. Therefore, the aim of this study was to investigate the antifungal effects of *C. longa, R. centifolia, R. officinalis*, and *P. granatum* glycolic extracts against the planktonic and biofilm forms of *C. albicans*, *C. dubliniensis, C. tropicalis*, and *C. krusei*. The null hypothesis is that, *C. longa, R. centifolia, R. officinalis*, and *P. granatum* glycolic extracts have no antifungal action against *Candida* spp.

## 2. Results

### 2.1. High-Performance Liquid Chromatography (HPLC) Analyses of Plant Extracts 

#### 2.1.1. *Rosmarinus officinalis* L. Glycolic Extract 

Gallotannins of 414.92 and 243.29 μg/100 mL were found at 11.09 and 13.05 min, respectively. Such compounds were quantified from the standard curve of gallic acid. Besides, chlorogenic acid of 126.99 μg/100 mL was found at 12.64 min, and p-coumaric acid of 2153.22 μg/100 mL at 14.83 min (Table 1).

#### 2.1.2. *Punica granatum* L. Glycolic Extract

Eight different gallotannins were identified, expressed as gallic acid, totaling a concentration of 4318.31 μg/100 mL of the glycolic extract. In addition, a compound of medium polarity was identified, which could be quercetin or kaempferol, at a concentration of 5233.16 μg/100 mL at 32.5 min (Table 2).

#### 2.1.3. *Rosa centifolia* L. Glycolic Extract 

Gallic acid of 946.6 μg/100 mL was found at 11.09 min, gallotannins of 628.1 and 648.6 μg/100 mL were found at 12.94 and 14.73 min, respectively, p-coumaric acid of 591.5 μg/100 mL was found at 15.75 min, and quercetin derivatives 3316.50 μg/100 mL were found at 37.71 min (Table 3).

#### 2.1.4. *Curcuma longa* L. Glycolic Extract 

Curcumin of 135.09 μg/100 mL was found at 24.68 min (Table 4).

### 2.2. Minimum Inhibitory Concentration (MIC) and Minimum Fungicidal Concentration (MFC) 

MIC and MFC values of the *R. centifolia*, *C. longa*, *R. officinalis*, and *P. granatum* glycolic extracts varied from 12.5 to 50 mg/mL, as shown in Table 1 and Table 2. The *C. longa* glycolic extract did not produce a fungicidal effect against *C. krusei* at the tested concentration (Table 5 and Table 6).

### 2.3. Combined Extracts’ Synergistic Effects

The combination of *C. longa* and *R. centifolia* glycolic extracts showed additive values for all tested *Candida* spp., as calculated by the fractional inhibitory concentration (FIC) index (Table 5). The combination of *R. officinalis* and *P. granatum* glycolic extracts showed additive values for all tested *Candida* spp., as calculated by the FIC index (Table 6). 

### 2.4. Antibiofilm Activity

#### 2.4.1. *C. albicans* Biofilms

**Figure 1:**
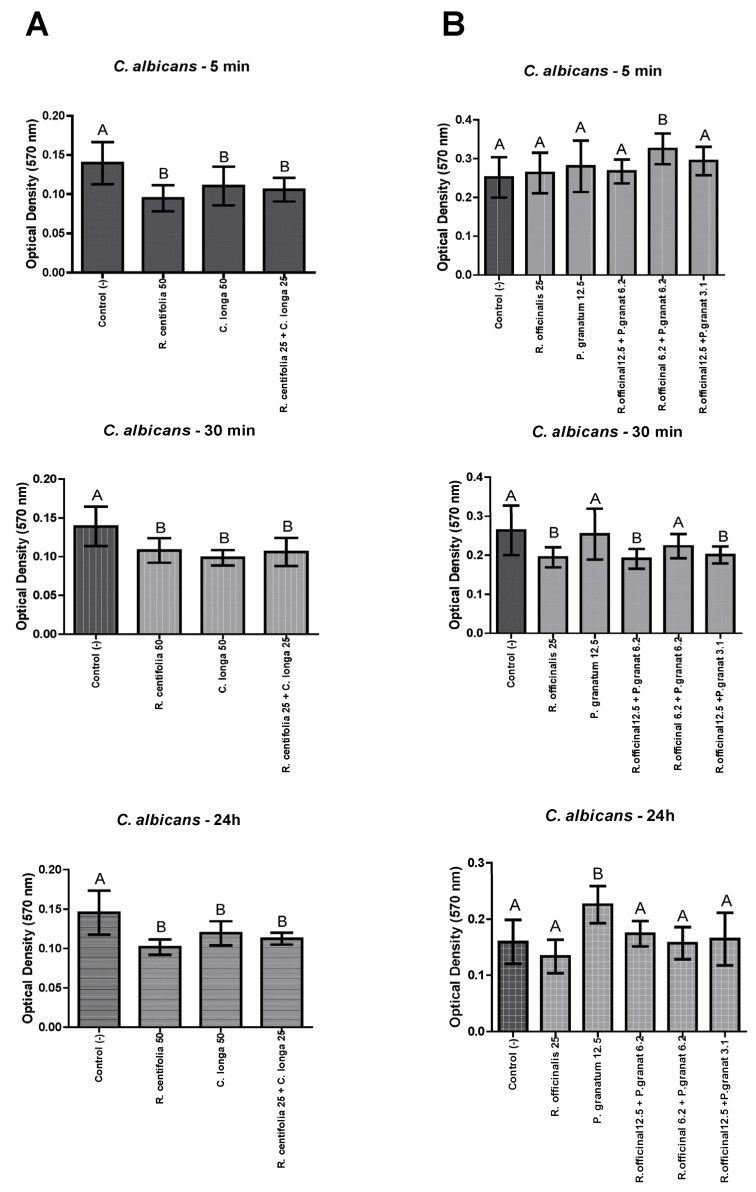
*C. albicans* biofilm load reduction after treatment with *R. centifolia*, *C. longa*, *R. officinalis*, and *P. granatum* glycolic extracts, and their combinations, for 5 min, 30 min, and 24 h. Legend: Different upper-case letters indicate a statistically significant difference.

*Rosa centifolia* and *C. longa* glycolic extracts, and their combination, were effective against *C. albicans* biofilms after 5 min, 30 min, and 24 h, with a statistically significant difference with the control group (*p* ≤ 0.05) (Figure 1A). Differently, only *R. officinalis* glycolic extract, *R. officinalis* 12.5 + *P. granatum* 6.2, and *R. officinalis* 12.5 + *P. granatum* 3.1, were effective against *C. albicans* biofilms after 30 min, with a statistically significant difference with the control group (*p* ≤ 0.05) (Figure 1B).

#### 2.4.2. *C. dubliniensis* Biofilms

**Figure 2:**
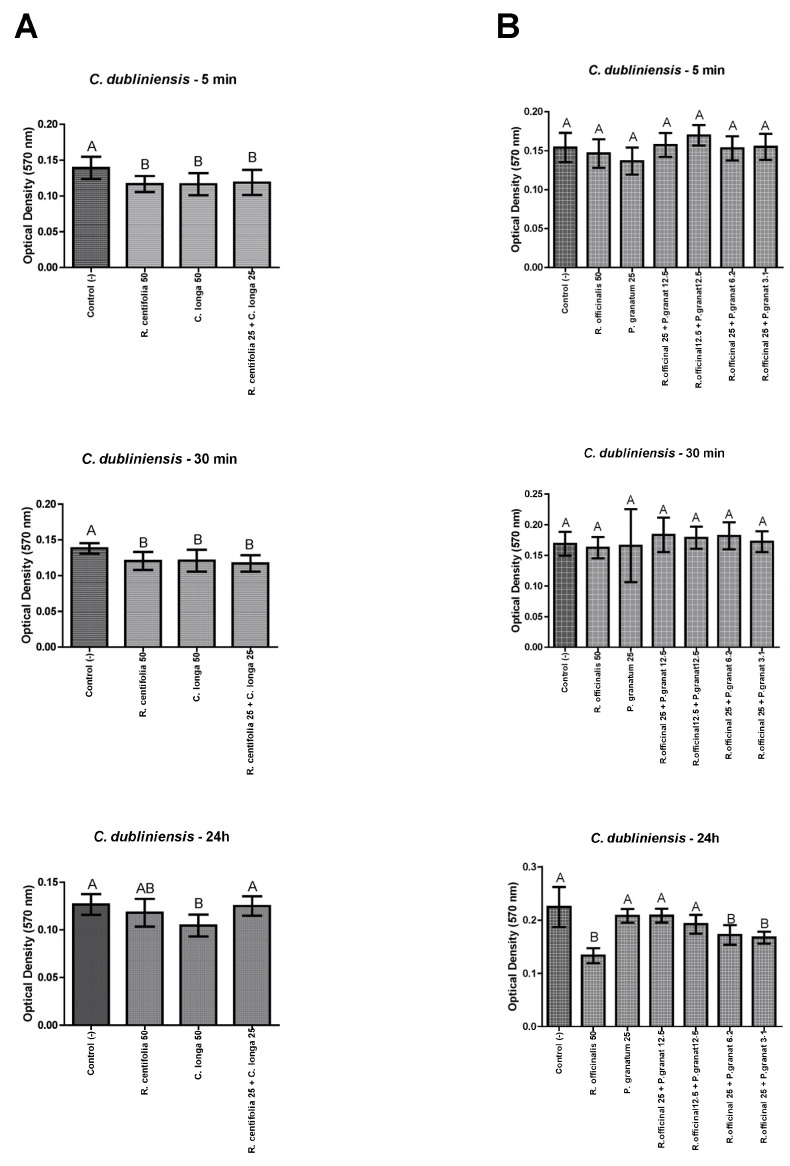
*C. dubliniensis* biofilm load reduction after treatment with *R. centifolia*, *C. longa*, *R. officinalis*, and *P. granatum* glycolic extracts, and their combinations, for 5 min, 30 min, and 24 h. Legend: Different upper-case letters indicate a statistically significant difference.

*Rosa centifolia* and *C. longa* glycolic extracts, and their combination, were effective against *C. dubliniensis* biofilms after 5 min and 30 min, however, only *C. longa* glycolic extract was effective after 24 h, all these comparisons had a statistically significant difference with the control group (*p* ≤ 0.05) (Figure 2A). Besides, only *R. officinalis* 25 + *P. granatum* 6.2, and *R. officinalis* 25 + *P. granatum* 3.1 were effective against *C. dubliniensis* biofilms after 24 h, with a statistically significant difference with the control group (*p* ≤ 0.05) (Figure 2B).

#### 2.4.3. *C. tropicalis* Biofilms

**Figure 3:**
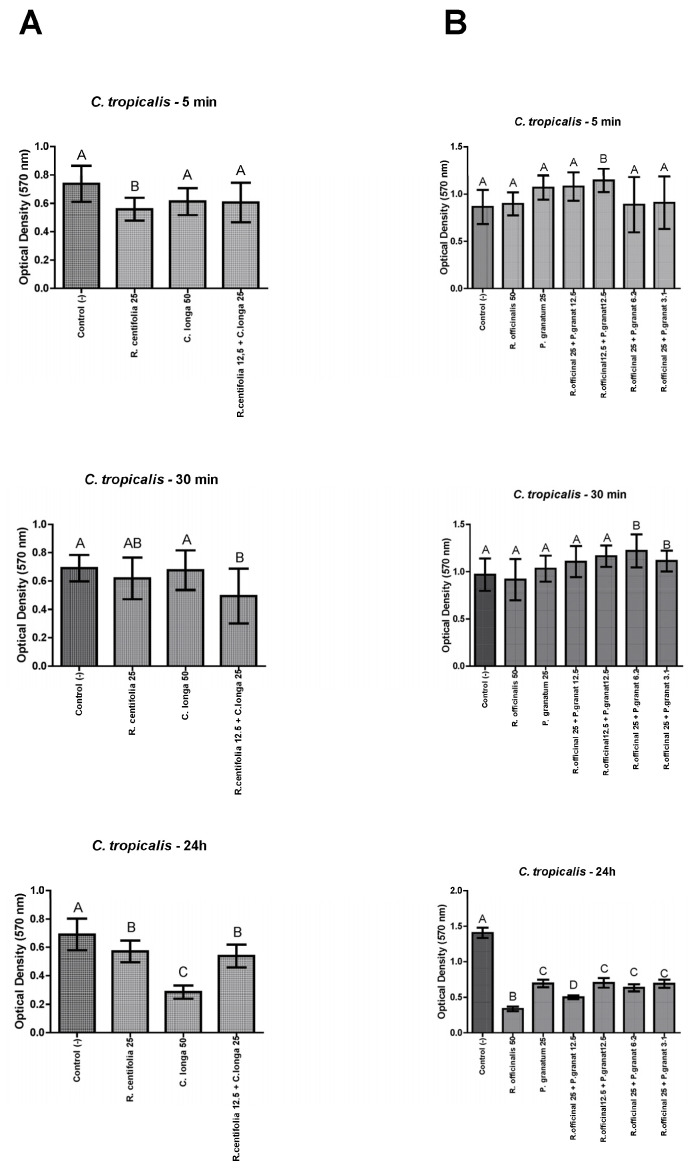
*C. tropicalis* biofilm load reduction after treatment with *R. centifolia*, *C. longa*, *R. officinalis*, and *P. granatum* glycolic extracts, and their combinations, for 5 min, 30 min, and 24 h. Legend: Different upper-case letters indicate a statistically significant difference.

*Rcentifolia* and *C. longa* glycolic extracts, and their combination, were effective against *C. tropicalis* biofilms after 24 h, however, after 30 min only their combination, and after 5 min only the *R. centifolia* glycolic extract were effective, with a statistically significant difference with the control group (*p* ≤ 0.05) (Figure 3A). Besides, *R. officinalis* and *P. granatum* glycolic extracts, and all their combinations, were effective against *C. tropicalis* biofilms after 24 h, with a statistically significant difference with the control group (*p* ≤ 0.05) (Figure 3B).

#### 2.4.4. *C. krusei* Biofilms

**Figure 4:**
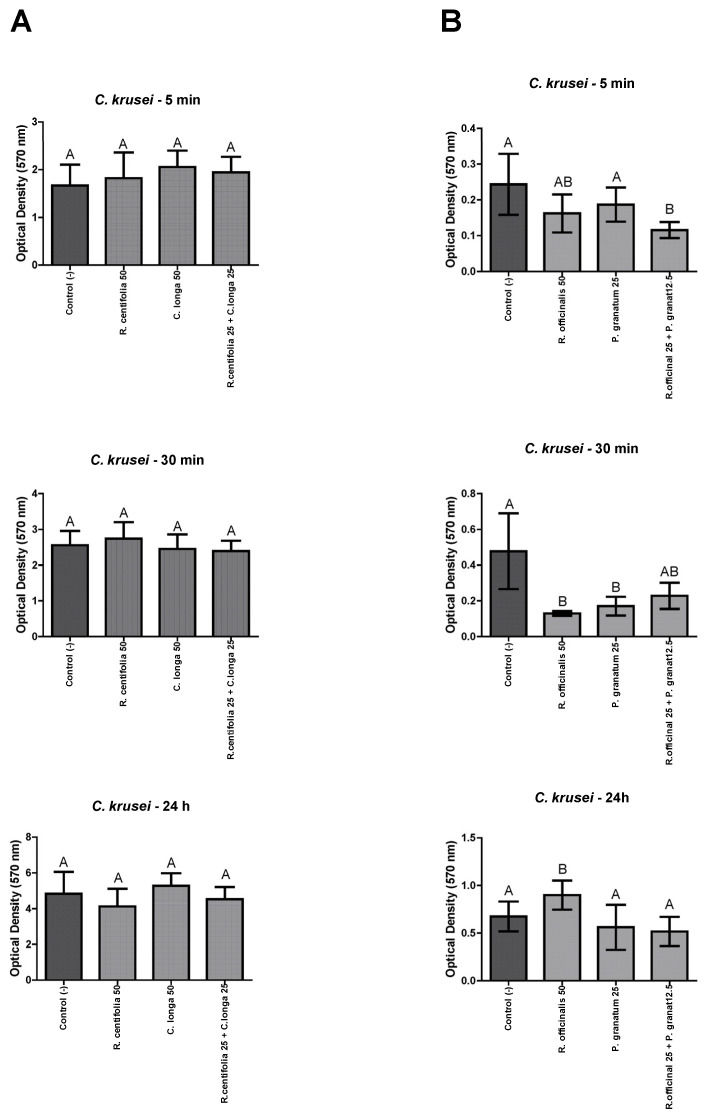
*C. krusei* biofilm load reduction after treatment with *R. centifolia*, *C. longa*, *R. officinalis*, and *P. granatum* glycolic extracts, and their combinations, for 5 min, 30 min, and 24 h. Legend: Different upper-case letters indicate a statistically significant difference.

*R centifolia* and *C. longa* glycolic extracts, and their combination, were not effective against *C. krusei* biofilms (*p* > 0.05) (Figure 4A). Conversely, *R. officinalis* and *P. granatum* glycolic extracts, and their combination, were effective against *C. krusei* biofilms after 30 min, being all statistically equal (*p* ≤ 0.05) (Figure 4B).

## 3. Discussion

Herbal medicines are increasingly used as alternative therapeutic agents [16,28,29]. The synergism of herbal medicines was described as a successful alternative to improve their antimicrobial action [30], to combat multi-drug resistant microorganisms [31]. This study was carried out to investigate the isolated and combined effects of *R. centifolia* + *C. longa* and *R. officinalis* + *P. granatum* glycolic extracts, against *C. albicans*, *C. dubliniensis, C. tropicalis*, and *C. krusei*. In this study, the isolated and combined extracts, in different concentrations, were effective against the tested *Candida* spp., therefore, the null hypothesis of this study was rejected.

Meccatti et al. (2021) [18], evaluated the chemical and biological properties of *R. officinalis* glycolic extract, and identified a derivative of chlorogenic acid at 12.61 min. In the present study, the same compound was also identified, with a similar retention time (12.64 min). Besides, in another study [32], the same extract was analyzed by HPLC/DAD under similar chromatographic conditions, and p-coumaric acid of 1.37 mg/100 g, a derivative of cinnamic acid, was found at 13.94 min, similarly, p-coumaric acid of 2153.22 µg/100 mL was identified in the present study, at 14.83 min. Gallotannins expressed in gallic acid were also identified at 11.09 and 13.05 min, in the same mentioned study, gallic acid was also identified in the *R. officinalis* extract at 6.65 min. 

Several phytocompounds identified in *R. officinalis* extracts and essential oils have pharmacological activities, and the concentration of these molecules varies according to the specimen and region of the plant. The most reported phytocompounds include carnosic acid, rosmarinic acid, and chlorogenic acid [24]. The chlorogenic acid, identified in our study, has antioxidant, antibacterial, and antifungal (against *Candida*) action [33,34], as it reduces cell viability, increases the potential for mitochondrial depolarization and production of reactive oxygen species, and causes DNA fragmentation in fluconazole-resistant *Candida* species [35]. 

In the present study, several gallotannins expressed in gallic acid were identified in the *P. granatum* extract, totaling a concentration of 4318.30 μg/100 mL, in addition, another compound, of medium polarity, of 5233.16 μg/100 mL, was identified at 32.5 min, which could be quercetin or kaempferol. In a study on phytochemical analysis of methanolic extracts of *P. granatum* peel, analyzed by HPLC [36], gallic acid of 7.89 mg/mL at 14.94 min, quercetin of 20.22 mg/mL at 20.32 min, p-coumaric acid 19.85 mg/mL at 12.32 min, and cinnamic acid of 31.69 mg/mL at 16.21 min, were identified. The different components, in comparison with the present study, may be explained by the different vehicle used in the other study. Gallic acid and polyphenols, such as tannins, prove the antifungal action of the *P. granatum* extract [37].

In the present study, a quercetin derivative, gallic acid, p-coumaric acid, and gallotannins were identified. In another study [38], chlorogenic acid and gallic acid were identified, which is in agreement with our study. Still, the present study seems to be a pioneer in identifying p-coumaric acid (591.5 μg/100 mL) in the *R. centifolia* glycolic extract. 

It is well-known that curcumin is the main phytocompound of *C. longa*. Still, the literature contains several studies on the application of curcumin as a promising photosensitizer to be applied in photodynamic therapy, including against *Candida* spp. [15]. In the present study, curcumin of 135.09 μg/100 mL was identified at 24.68 min. For Sabir et al. (2021) [39], curcumin, as 3202.9 µg/g of the dry weight of the ethanolic extract of the rhizome, was identified at 35 min. 

In the microbiological analyses, it was found that the MIC and MFC values of the *R. centifolia, C. longa, R. officinalis*, and *P. granatum* glycolic extracts varied between 12.5–50 mg/mL, furthermore, it was noticed that lower concentrations (12.5 mg/mL) of the *P. granatum* glycolic extract were as effective as the greater concentrations of the other extracts. In the literature, the antifungal action of the four tested extracts was proven against diverse *Candida* spp. in different study models [15,18,23,26].

*R centifolia* was tested at 200 mg/mL against diverse oral microbes, using the inhibition zone test, and it was found to be effective against *S. mutans, C. albicans*, and *C. tropicalis* [20]. In the present study, *R. centifolia* glycolic extract at 50 mg/mL was also effective against *C. albicans*, *C. dubliniensis,* and *C. tropicalis* after 5 min, 30 min, and 24 h.

*C longa* methanolic and ethanolic extracts were effective against *C. albicans* at 5–100 mg/mL after 24 h, evaluated by agar disc diffusion method [40], differently, *C. longa* alcoholic extract at 200 mg/mL was tested as an endodontic irrigant, and was not as effective as sodium hypochlorite after 24 h [41], the same alcoholic extract was effective against *C. albicans* in four different dilutions [42]. In more recent studies, the antifungal effect of *C. longa* was shown to be as effective as nystatin [15,43].

In the present study, when *R. centifolia* and *C. longa* glycolic extracts were combined, it was found that the MIC of each extract was reduced by a factor of two. The additive effect resulting from this combination of the extracts, against *C. albicans*, *C. dubliniensis*, *C. tropicalis*, and *C. krusei*, as FIX index, was between 0.5 and 1.0. In biofilms, the combined treatment presented the best results after 30 min against *C. albicans, C. dubliniensis,* and *C. tropicalis*, however, it was not active against *C. krusei*, as *C. krusei* is characterized by resistance against fluconazole in more than 95% of clinical and veterinary isolates [44]. To the best of our knowledge, this is the first study that has evaluated the synergistic effect of *R. centifolia* and *C. longa* glycolic extracts, making the results of this study pioneering. In the literature, the combined effect of turmeric oil with antifungal drugs was analyzed against *C. albicans* and *Trichophyton mentagrophytes*, this combination increased the drugs’ antifungal activity [45].

*Rosmarinus officinalis* essential oil and methanolic extract were introduced in the literature as effective antibacterial and antifungal agents [46,47,48]. Furthermore, it was indicated as being a prophylactic antifungal agent at 6.25 mg/mL for 72 h and 12.5 mg/mL for 24 h, in a *Galleria mellonella* model study [17]. In the present study, *R. officinalis* glycolic extract was effective against *C. albicans* biofilms after 30 min, and against *C. dubliniensis* biofilms after 24 h, at 50 mg/mL. *P. granatum* has also been reported to be an effective antibacterial and antifungal agent [49,50], and especially against resistant strains [51]. In the present study, it was effective at 25 mg/mL only against *C. tropicalis*, after 24 h, and against *C. krusei* after 30 min. 

Interestingly, the combination of *R. officinalis* and *P. granatum* glycolic extracts reduced the MIC by up to a factor of eight. One additive combination was found to have activity against *C. krusei*, three against *C. albicans,* and four against the other non-*albicans* species. In the study by Abu El-Wafa et al. [52], some plant extracts, isolated and in combination with antibiotics, were tested, including *R. officinalis* and *P. granatum*, it was found that the rosemary and pomegranate extracts together showed synergism (FIC index < 0.5) with the antibiotics (piperacillin, ceftazidime, imipenem, gentamicin, or levofloxacin), in which the plant extracts significantly reduced the MIC values against a clinical strain of *Pseudomonas aeruginosa*.

It was found in the present study, that *C. albicans* biofilms suffered a significant reduction when the isolated *R. officinalis* extract, and the combinations *R. officinalis* 12.5 + *P. granatum* 6.2 and *R. officinalis* 12.5 + *P. granatum* 3.1, were applied. Furthermore, the combination *R. officinalis* 25 + *P. granatum* 12.5 was effective against *C. krusei*. For Abu El-Wafa et al. (2020) [52], the combination of rosemary, pomegranate, and antibiotic, achieved up to 99.6% eradication of a *P. aeruginosa* biofilm, presenting the best results when compared to any other monotherapy evaluated in the study.

Further, *R. officinalis* extract, and the combinations *R. officinalis* 25 + *P. granatum* 3.1 and *R. officinalis* 25 + *P. granatum* 6.2, promoted significant reductions in *C. dubliniensis* biofilms after 24 h. It seems like that a prolonged contact with *C. dubliniensis* biofilms resulted in the highest percentages of biofilm reduction. A significant reduction in *C. tropicalis* biofilms was also observed with all tested groups after 24 h. Similarly, in another study, the prolonged time of incubation enabled the red propolis to interfere in the formation of the biofilms of *C. albicans, C. tropicalis and C. dubliniensis* [53].

The present work has presented important findings on the antifungal action and chemical compositions of plant extracts, revealing different additive combinations with exploitable potential for the formulation of new antifungal compounds as endodontic irrigant, intracanal medication, mouthwash, or toothpaste components. We also characterized and quantified chemical compounds such as chlorogenic acid, quercetin, gallic acid, p-coumaric acid, and curcumin in the glycolic extracts. For future analyses, we envision the possibility of developing several compounds to treat and aid in the fight against oral candidiasis, such as the development of a plant-based ointment, that presents substantivity for application on the affected mucous membranes, or even the development of a disinfectant solution for dental prostheses, that would be soaked for 30 min a day as an adjuvant method of cleaning.

## 4. Materials and Methods

### 4.1. Plants Extracts

Four extracts were obtained: *C. longa* rhizomes (Seiva Brazilis, SP, Brazil), *R. centifolia* flowers, *R. officinalis* leaves, and *P. granatum* bark (Mapric^®^, São Paulo, SP, Brazil). All extracts were not manipulated in our laboratory; however, they were obtained commercially with reports and specifications from the manufactures. The extracts were formulated at a concentration of 20% (200 mg of the extract powder) and were diluted in 1 mL of propylene glycol (Mapric^®^, São Paulo, SP, Brazil). The use of plant parts in the present study complies with international, national, and/or institutional guidelines.

### 4.2. High-Performance Liquid Chromatography (HPLC) Analyses of Plant Extracts 

High-performance liquid chromatography (HPLC) was used to characterize and quantify the contents of markers in the natural extracts. The analysis was carried out in a high-efficiency liquid chromatograph, with a diode-array detection (HPLC-DAD) and an automatic injector, model D-7000 Merck-Hitachi (Merck KGaA, Darmstadt, Germany). The chromatographic conditions were, mobile phase: water–formic acid solution (PA, Merck), diluted in the ratio of 95:5 (solvent A), and methanol HPLC grade Merck (Merck KGaA, Darmstadt, Germany, solvent B). The flow was 1 mL/min, and a linear gradient, starting with 0% B, ending with 70% B, in a 50 min run time. The detection wavelengths were 280 and 340 nm [54].

### 4.3. Construction of Standard Curves

The stock solutions of the standards referring to the main identified compounds were prepared in methanol, at different concentrations. A standard curve was constructed using these solutions. Aliquots of these solutions were filtered through 0.45 µm membrane filters (Millipore, Thermo Fisher Scientific, São Paulo, SP, Brazil) for HPLC analysis. The straight-line equation of each of the calibration curves was used to confirm and quantify the compounds found in the plant extracts. Quantification was performed using standards with the same ultraviolet spectrum.

### 4.4. Inoculum Preparation 

The reference strains (ATCC, American Type Culture Collection, Rio de Janeiro, RJ, Brazil) of *C. albicans* ATCC 18804, *C. dubliniensis* ATCC MYA 646, *C. tropicalis* ATCC 13803, and *C. krusei* ATCC 6258 were used in this study. They were grown in Sabouraud Dextrose (SD) Agar (Kasvi, São José dos Pinhais, PR, Brazil) at 37 °C for 24 h, then supplemented with chloramphenicol 50 mg/L (Union Química, Belo Horizonte, MG, Brazil). Standardized inoculum of each strain was prepared and adjusted using a spectrophotometer (Micronal B-582, Micronal, São Paulo, SP, Brazil) at 530 nm and an optical density of 0.284, in a concentration of 1 × 10^6^ yeast cells per milliliter, as detailed in a previous study [15].

### 4.5. Minimum Inhibitory (MIC) and Minimum Fungicidal (MFC) Concentrations 

A microdilution method was used, according to Clinical and Laboratory Standards Institute (CLSI) documents M27–S4, to determine the minimum inhibitory (MIC) and minimum fungicidal (MFC) concentrations of the glycolic extracts for each fungal strain. In different 96-well microplates (TPP, Trasadingen, Switzerland) for each fungal strain, 100 µL/well of RPMI 1640 (INLAB, São Paulo, Brazil), with glutamine, without bicarbonate and with phenol red indicator, was added. Next, 200 µL of one of the glycolic extracts was added in the first well, where 10 serial dilutions were carried out. Then, 100 µL/well of the respective fungal inoculum was added. The microplates were incubated for 48 h at 37 °C. The MIC of each glycolic extract was determined, by eye, in the first well without turbidity after the well with apparent microbial growth. In addition, analyses of the vehicle (propylene glycol) were performed to verify if it interferes with the antimicrobial action of plant glycolic extracts. To determine the MFC, the contents of the well where the MIC was determined and the previous well were seeded onto SD agar (Kasvi, São José dos Pinhais, PR, Brazil). After incubation (37 °C/48 h), the MFC was determined as the lowest concentration of the glycolic extract that inhibited total microorganism growth.

### 4.6. Combined Extracts’ Synergistic Effects

The chessboard technique was used, according to Moreno et al. [55], with modifications, this method is based on the microdilution method of the Clinical and Laboratory Standards Institute. Concentrations of each glycolic extract were prepared, starting with MIC (MIC, MIC/2, MIC/4, MIC/8, MIC/16, MIC/32, and MIC/64). For this purpose, a serial dilution of the first glycolic extract (*R. centifolia* or *R. officinalis*) was carried out along the x-axis (horizontal) of the microplate. The dilution of the second glycolic extract (*C. longa* or *P. granatum*) was carried out separately and added to the y-axis (vertical) of the microplate. In this way, control of concentrations was maintained. Saline solution and culture medium were used as control groups. Finally, 100 µL of standardized inoculum (1 × 10^6^ cells/mL) of each strain was added, totaling 200 µL/well.

The microplates were incubated at 37 °C/48 h, for further visual reading. To evaluate the synergistic effect of the glycolic extracts, the fractional inhibitory concentration (FIC) index was adopted, which classifies the combinations as synergistic, additive, indifferent, or antagonistic. The FIC index was calculated using the formula:

FIC index = FIC1st + FIC2nd = (MIC of the 1st extract in combination/MIC of the 1st extract alone) + (MIC of the 2nd extract in combination/MIC of the 2nd extract alone).

The combination was considered synergistic when FIC ≤ 0.5, additive when FIC > 0.5 and ≤1.0, indifferent when FIC > 1 and ≤4, and antagonist when FIC > 4.0. The same formula was used to calculate the fractional fungicide concentration index (FFC), using MFC values instead of MIC [55].

### 4.7. Antibiofilm Activity by MTT Assay

Based on the studies by Meccatti et al. [18] and de Sá Assis et al. [54], the inoculum was standardized in a spectrophotometer to obtain 10^7^ cells/mL. Subsequently, 200 µL/well of fungal inoculum was added to microplates and incubated for 90 min at 37 °C, to permit initial adhesion of fungal cells to the well. Then, the supernatant was discarded and BHI (Brain Heart Infusion—Kasvi, São José dos Pinhais, PR, Brazil) broth was added. Incubation continued for 48 h for biofilm formation, with the culture medium being changed after 24 h of incubation.

After biofilm formation, MIC values of sole extracts and additive concentrations of the combined extracts were applied for 5 min, 30 min, and 24 h. Culture medium was used as a negative control. Each experimental group consisted of n = 10. After the exposure period, saline solution was added and this was discarded to wash the wells and eliminate non-adherent cells that suffered from the treatment and detached from the bottom of the well. Then, 100 µL of MTT (3-(4,5-dimethylthiazol-2-yl)-2,5-diphenyltetrazolium bromide) solution (Sigma-Aldrich, Brazil) was added in each well and the plate was incubated in the dark at 37 °C for 1 h. Then, the MTT solution was removed and 100 µL of dimethylsulfoxide (DMSO) was added. The plates were again incubated at 37 °C for 10 min and agitated in a shaker, under constant agitation, for 10 min. The optical densities (OD) were obtained using a microplate reader at 570 nm, and the OD obtained were converted into percentage of the metabolic activity of the fungal cells.

### 4.8. Statistical Analysis

Data were analyzed by normality tests: Shapiro–Wilk, Kolmogorov–Smirnov and D’Agostino and Pearson omnibus, and for homogeneity using the Bioestat 5.0 software. Normal data were analyzed by ANOVA and Tukey’s test, otherwise, by the Kruskal–Wallis test and Dunn’s test. The GraphPad Prism 5.0 program was used, considering a significance level of 5%. 

## 5. Conclusions

The main phytochemical compounds in the extracts were identified and quantified, and the present study is a pioneer in reporting the presence of gallotannins and p-coumaric acid in the *R. centifolia* extract.

The combined plant extracts showed antifungal and antibiofilm action against *Candida* spp., and in some protocols, such as the application of *R. officinalis* and *P. granatum* for 30 min against *C. krusei,* and 24 h against *C. tropicalis*, average reductions of 50% in biofilm were found.

Such results bring to light the possibility of developing compounds for the treatment of, and to aid in the fight against, oral candidiasis, such as the development of a vegetable-based ointment that presents substantivity for application on the affected mucous membranes, or even the development of a disinfectant solution for dental prostheses. New pharmacodynamic studies, to standardize combinations, and animal studies, must be performed to advance product development.

## Figures and Tables

**Table 1:** *Rosmarinus officinalis* L. glycolic extract characterization.

Retention Time (min)	Compound Name	Quantity (μg/100 mL)
11.09	gallotannin *	414.92
13.05	gallotannin *	243.29
12.64	chlorogenic acid	126.99
14.83	p-coumaric acid **	2153.22

* The same ultraviolet spectrum as gallic acid, with different retention time. Expressed as gallic acid. ** The same ultraviolet spectrum as p-coumaric acid, with different retention time. Expressed as p-coumaric acid.

**Table 2:** *Punica granatum* L. glycolic extract characterization.

Retention Time (min)	Compound Name	Quantity (μg/100 mL)
11.07	gallotannin *	122.3
12.84	gallotannin *	936.3
14.81	gallotannin *	955.1
17.11	gallotannin *	825.7
18.55	gallotannin *	405.9
20.21	gallotannin *	94.4
25.49	gallotannin *	386.8
32.5	quercetin or kaempferol ** (medium polarity)	5233.16

* The same ultraviolet spectrum as gallic acid, with different retention time. Expressed as gallic acid. ** The same ultraviolet spectrum as quercetin, with different retention time. Expressed as quercetin.

**Table 3:** *Rosa centifolia* L. glycolic extract characterization.

Retention Time (min)	Compound Name	Quantity (μg/100 mL)
11.09	gallic acid	946.6
12.94	gal *	628.1
14.73	gal *	648.6
15.75	p-coumaric acid **	591.5
37.71	derivative of quercetin ***	3316.50

* The same ultraviolet spectrum as gallic acid, with different retention time. Expressed as gallic acid. ** The same ultraviolet spectrum as p-coumaric acid, with different retention time. Expressed as p-coumaric acid. *** The same ultraviolet spectrum as quercetin, with different retention time. Expressed as quercetin.

**Table 4:** *Curcuma longa* L. glycolic extract characterization.

Retention Time (min)	Compound Name	Quantity (μg/100 mL)
24.68	curcumin	135.09

**Table 5:** Minimum inhibitory concentration (MIC) and minimum fungicidal concentration (MFC) values in mg/mL, in addition to the combined effect of *R. centifolia* and *C. longa* glycolic extracts, against *Candida* spp.

*Candida* spp.	Isolated Extract MIC and MFC Value (mg/mL)		Combined Concentrations (mg/mL)		FIC Index	Reduction in MIC		Effect
	*R. centifolia*	*C. longa*	*R. centifolia*	*C. longa*		*R. centifolia*	*C. longa*	
*C. albicans*	50	50	25	25	1.00	2×	2×	Add
*C. dubliniensis*	50	50	25	25	1.00	2×	2×	Add
*C. tropicalis*	25	50	12.5	25	1.00	2×	2×	Add
*C. krusei*	50	50	25	25	1.00	2×	2×	Add
			12.5	25	0.75	2×	4×	Add

Legend: Add: additive effect. FIC index = FIC1st + FIC2nd = (MIC of the 1st extract in combination/MIC of the 1st extract alone) + (MIC of the 2nd extract in combination/MIC of the 2nd extract alone). The results were interpreted as synergistic (≤0.5), additive (>0.5 and ≤1.0), indifferent (>1.0 and <4.0), or antagonist effect (≥4.0) (Moreno et al., 2020).

**Table 6:** Minimum inhibitory concentration (MIC) and minimum fungicidal concentration (MFC) values in mg/mL, in addition to the combined effect of *R. officinalis* and *P. granatum* glycolic extracts, against *Candida* spp.

*Candida* spp.	Isolated Extract MIC and MFC Value (mg/mL)		Combined Concentrations (mg/mL)		FIC Index	Reduction in MIC		Effect
	*R. officinalis*	*P. granatum*	*R. officinalis*	*P. granatum*		*R. officinalis*	*P. granatum*	
*C. albicans*	25	12.5	12.5	6.2	0.9	2×	2×	Add
			6.2	6.2	0.6	4×	2×	Add
			12.5	3.1	0.7	2×	4×	Add
*C. dubliniensis*	50	25	25	12.5	1.0	2×	2×	Add
			12.5	12.5	0.7	4×	2×	Add
			25	6.2	0.7	2×	4×	Add
			25	3.1	0.6	2×	8×	Add
*C. tropicalis*	50	25	25	12.5	1.0	2×	2×	Add
			12.5	12.5	0.7	4×	2×	Add
			25	6.2	0.7	2×	4×	Add
			25	3.1	0.6	2×	8×	Add
*C. krusei*	50	25	25	12.5	1.0	2×	2×	Add

Legend: Add: additive effect. FIC index = FIC1st + FIC2nd = (MIC of the 1st extract in combination/MIC of the 1st extract alone) + (MIC of the 2nd extract in combination/MIC of the 2nd extract alone). The results were interpreted as synergistic (≤0.5), additive (>0.5 and ≤1.0), indifferent (>1.0 and <4.0) or antagonist effect (≥4.0) (Moreno et al., 2020).

## Data Availability

The data used to support the findings of this study are available upon request from the corresponding author, d.d.s.amjad@gmail.com.

## Sample Availability

The samples of this study are available upon request from the corresponding author, d.d.s.amjad@gmail.com.
